# Chromatin changes trigger laminin genes dysregulation in aging kidneys

**DOI:** 10.18632/aging.101453

**Published:** 2018-05-29

**Authors:** Oleg Denisenko, Daniel Mar, Matthew Trawczynski, Karol Bomsztyk

**Affiliations:** 1 Department of Medicine, University of Washington, Seattle, WA 98109, USA

**Keywords:** DNA methylation, epigenetics, gene expression, laminin, aging kidney

## Abstract

Dysregulation of gene expression is a hallmark of aging. We examined epigenetic mechanisms that mediate aberrant expression of laminin genes in aging rat kidneys. In old animals, no alterations were found in the levels of abundant laminin mRNAs, whereas Lama3, b3, and c2 transcripts were increased compared to young animals. Lamc2 showed the strongest changes at the mRNA and protein levels. Lamc2 upregulation was transcriptional, as indicated by the elevated RNA polymerase II density at the gene. Furthermore, aging is associated with the loss of H3K27m3 and 5mC silencing modifications at the Lamc2 gene. Western blot analysis revealed no changes in cellular levels of H3K27m3 and cognate enzyme Ezh2 in old kidneys. Thus, the decrease in H3K27m3 at Lamc2 resulted from the re-distribution of this mark among genomic sites. Studies in kidney cells *in vitro* showed that reducing H3K27m3 density with Ezh2 inhibitor had no effect on Lamc2 expression, suggesting that this modification plays little role in gene upregulation in aging kidney. In contrast, treatment with DNA methylation inhibitor 2'-deoxy-5-azacytidine was sufficient to upregulate Lamc2 gene. We suggest that the loss of 5mC at silenced laminin genes drives their de-repression during aging, contributing to the age-related decline in renal function.

## Introduction

In organisms as diverse as yeast and humans, age-related changes in chromatin structure contribute to alterations in gene expression and progression to aging phenotypes [1]. The extracellular matrix (ECM) defines tissue compartments and orchestrates organ development and function [2]. ECM structure and function are altered with aging [3,4]. Specifically, one of the hallmarks of aging kidneys is the aberrant accumulation of ECM proteins in the interstitium (interstitial fibrosis) [5,6]. These alterations contribute to the age-related decline of kidney function, culminating in the organism’s death. Previously we have shown that, during aging, transcription of the ECM gene Col3a1 is increased in rat kidneys, a finding associated with aberrant accumulation of collagen III protein in the interstitium [7].

Laminins, along with collagens, are ECM proteins abundant in basement membranes [8,9]. Laminins are heterotrimer proteins consisting of α, β, and γ chains (one of each) [10]. In mammals, there are five α, three β and three γ chains, each encoded by its own gene. These chains assemble into 16 known laminin isoforms, and several of them have tissue-specific distribution [11]. Balanced temporal-spatial expression of specific laminin isoforms is important in maintaining organ architecture and function [8]. In kidneys, laminins are essential to the structure of the glomerular basement membrane (GBM), mesangial matrix (MM) and tubular basement membrane (TBM) [12]. The integrity of these membranes, in turn, is important for efficient blood filtration, nutrient re-absorption, solute homeostasis and waste removal. Some age-related changes in laminin chain B1 and s-laminin (encoded by Lamb1 and b2 genes) proteins were reported in the GBM [5], but to our knowledge, an analysis of changes in expression of all laminin chains in aging kidneys has not been done until now.

Although it is well established that aging *in vivo* and senescence *in vitro* trigger epigenetic changes that alter gene expression patterns [1,13], few studies have examined the contribution of epigenetic changes to age-related deregulation of ECM genes whose products maintain normal organ architecture. Here, we used a rat model of aging to elucidate changes in the epigenetic status of laminin chain genes in old kidneys.

## RESULTS

### Expression of laminin genes in young and old kidneys

To distinguish between common versus genotype-specific changes in laminin gene expression during aging, we used F344 and FBN-F1 rat lines, two established model systems supported by the National Institute of Aging (NIA). As life spans of these two rat lines are substantially different, we compared young animals (4 months old, 4 mo) to animals at the age corresponding to their median life span (50% survival), which is close to 24 months for F344 rats and 32 months for FBN-F1 rats [14]. 28 mo F344 rats (10% survival) were also used to examine progression of changes with aging.

**Figure 1:**
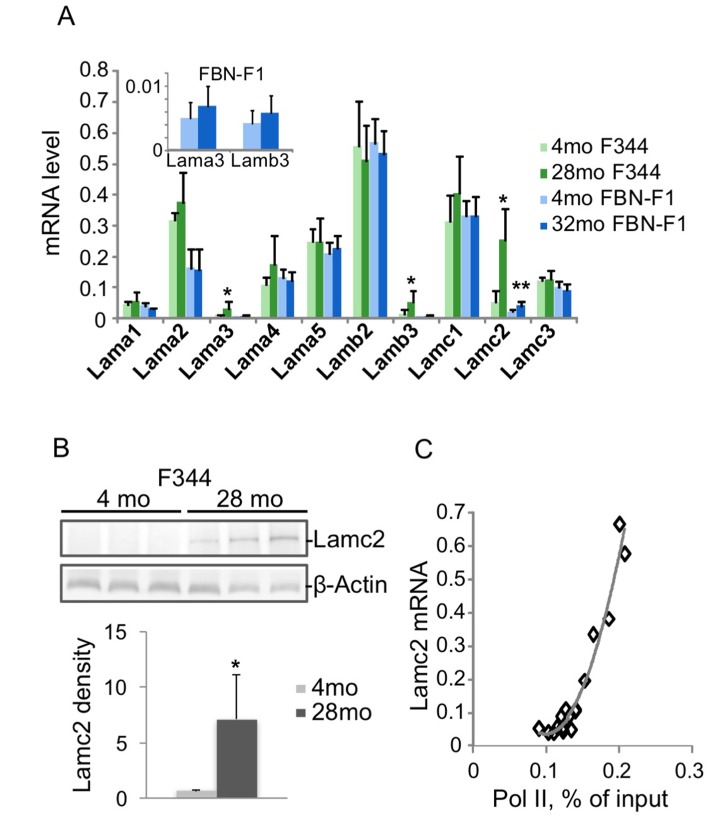
**Changes in laminin gene expression associated with aging in rat kidneys.** RNA was extracted from kidney fragments of 4 and 28 months old (mo) F344, and 4 and 32 mo FBN-F1 animals. Equal amounts of each RNA preparation were reverse transcribed and analyzed by qPCR with primers to laminin genes. Transcript levels were normalized to Gapdh mRNA. (**A**) Laminin mRNA levels were averaged per age group. Bars represent mean +SD, n=6 per age group. Inset, Lama3 and b3 mRNA levels in FBN-F1 animals shown at different scale. *p<0.05, **p<0.005. (**B**) Western blot analysis of Lamc2 levels in kidney extracts. β-Actin antibodies were used as a control. Densitometry analysis of blots is shown in the graph below as Lamc2/β-actin band ratio, mean +SD. (**C**) Pol II levels at Lamc2 gene were examined by ChIP assay with primers to the promoter of Lamc2 gene (-200 bp). Pol II density, expressed as a percent of input, is plotted against Lamc2 mRNA level. All age groups are shown.

The results of RT qPCR analysis of laminin transcript levels in F344 and FBN-F1 rat kidneys are shown in Figure 1A. Lama2, a4, a5, b2, c1, and c3 were highly expressed in young animals, with no change in old animals. In contrast, laminin genes expressed at very low levels in young animals – Lama3, b3, and c2 – were induced in old animals, with the largest changes seen in the Lamc2 transcript. Increased expression of Lamc2 in old F344 kidneys was also clearly detectable at the protein level (Figure 1B). The increase of Lamc2 in aging kidneys was associated with a matching increase in the density of RNA polymerase II (Pol II) recruited to the gene (Figure 1C), implicating transcriptional activation. Next, we explored the possibility of epigenetic dysregulation driving increased Lamc2 transcription.

### Increases in laminin transcript levels in aging kidneys are matched by decreases in the level of H3K27m3 histone modification at the corresponding genes

**Figure 2:**
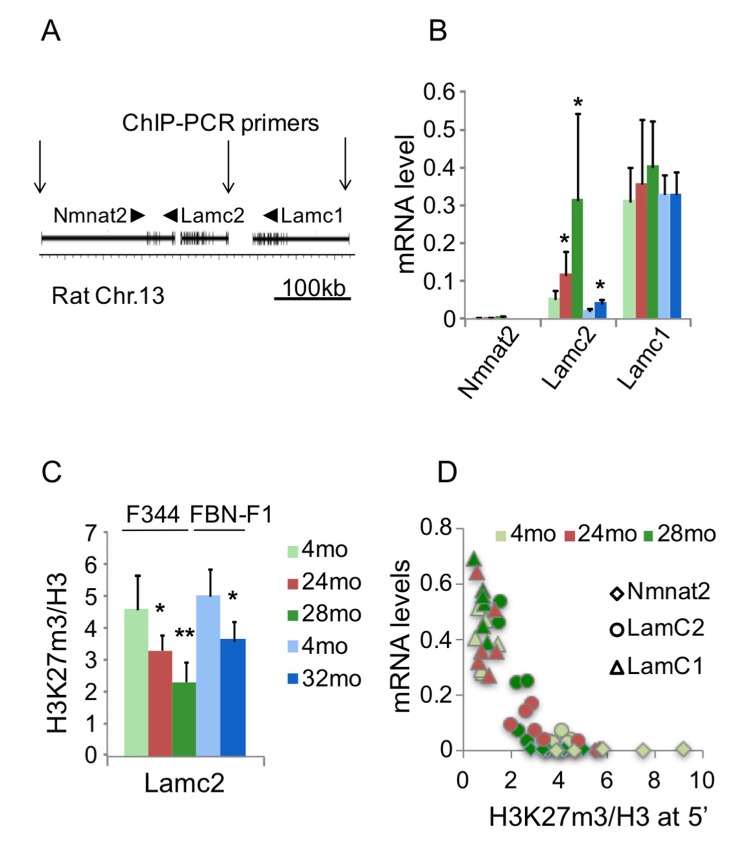
**Changes in transcription and H3K27m3 levels at the Lamc1-Lamc2 genomic region.** (**A**) A cartoon showing Nmnt2-Lamc2-Lamc1 genomic region. Arrows indicate position of 5’ PCR primer pairs used in ChIP assay. (**B**) Nmnat2, Lamc2 and Lamc1 transcript levels in 4, 24, 28 mo F344 and 4, 32 mo FBN-F1 kidneys. (**C**) ChIP analysis of H3K27m3 levels normalized to H3 at Lamc2 promoter in F344 and FBN-F1 young and old kidneys. Shown are mean values +SD, n=6 per age group, *p<0.05, **p<0.005. (**D**) H3K27m3 levels at the promoters of Nmnat2, Lamc2, and Lamc1 genes are inversely correlated with corresponding transcript levels. ChIP and RT PCR analyses were done in F344 kidney fragments. H3K27m3 data were normalized to H3 levels, and mRNA levels measured by RT PCR were normalized to Gapdh transcript levels. Each data point represents an individual animal. All age groups are shown.

To define the role of epigenetic processes in the deregulation of laminin gene expression in aging kidneys, we used Matrix ChIP assay [15] with antibodies to several histone modifications. Rat Lamc2 gene is located on chromosome 13 between Nmnat2 and Lamc1 genes (Figure 2A). Within this genomic region, Lamc1 gene is well expressed in young kidneys and shows no changes with aging. In contrast, Lamc2 is a low-abundance transcript in young kidneys, and its expression is significantly induced in old kidneys independent of genetic background (Figure 1A and 2B). Nmnat2 gene expression was not detected in kidneys of any age group (Figure 2B). Previous analysis showed that in different cell types and tissues, Lamc1 gene contains active histone marks, whereas Nmnat2 and Lamc2 reside within a silenced domain [16]. Thus, the Nmnat2-Lamc2-Lamc1 locus seems to be an attractive model of aging-associated chromatin changes in sections of the mammalian genome containing arrays of neighboring silenced and active domains.

**Figure 3:**
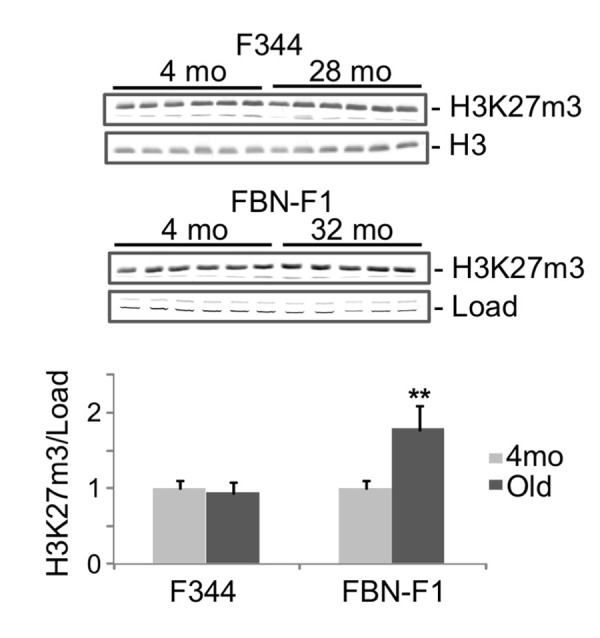
**Western blot analysis of H3K27m3 levels in histones extracted from young and old kidneys**. Upper panel, equal amounts of acid-extracted proteins from nuclear-enriched tissue fraction were subjected to SDS PAGE in 13% gels, transferred to PVDF membranes and stained with anti-H3K27m3 antibodies. Representative parts of ponseau-stained membranes are also shown (Load). Lower panel, results of densitometry analysis of bands shown in the upper panel. Y, young (4mo), O, old (28mo for F344, 32mo for FBN-F1). Signal intensities were normalized to load. Shown are mean values +SD, **p<0.005.

ChIP analysis of histone modification profiles revealed little or no changes in H3K4m3 density at Lamc1, Lamc2 and other laminin genes in old compared to young kidneys (not shown). There were also some decreases in H3K9m3, H3K36m3, and H3K79m2 levels along all tested loci (including laminin and control genes) which were not correlated with changes in corresponding transcript levels (not shown). It has previously been reported that aging is associated with the loss of silencing mark H3K27m3 at selected gene loci [17]. In agreement with these observations, the density of this mark was substantially decreased in old kidneys at the induced Lamc2 gene (Figure 2C). Similar decreases were found in both rat lines, indicating that this effect is not strain-specific (Figure 2C, compare 24mo F344 to 32mo FBN-F1). These observations suggest that the loss of H3K27m3 and the upregulation of Lamc2 gene were causally-related events in aging kidneys. Nuclear levels of H3K27m3 modification, as estimated by Western blot analysis, were not altered in F344 and were even increased in FBN-F1 old compared to young kidneys (Figure 3) showing that the loss of this histone mark was gene-specific rather than global.

Given the genomic proximity of Nmnat2, Lamc2 and Lamc1 genes, we examined the relationship between the mRNA expression level and H3K27m3 density at their gene promoters (Figure 2D). Interestingly, this analysis revealed that, at H3K27m3 density above a certain “threshold” level, no transcript could be detected. In contrast, below this threshold, transcript levels were inversely proportional to the H3K27m3 density.

### Changes in Ezh2 protein

Ezh2, a component of PRC2 complex, is a histone methyltransferase (KMT6A) for H3K27 [18]. It has been reported that cellular levels of Ezh2 protein decrease during aging *in vivo* and senescence *in vitro* [17,19,20]. In contrast, we have previously shown that in old F344 rat kidneys, levels of Ezh2 mRNA and protein were higher than in young animals [7]. Western blot analysis confirmed these observations in F344 kidneys (Supplementary Figure S1). In longer-lived FBN-F1 animals, there was no difference in the cellular levels of Ezh2 between old and young kidneys (Supplementary Figure S1). Other factors are likely involved in the loss of H3K27m3 at selected genes in aging kidneys.

We wondered if the loss of H3K27m3 at the Lamc2 gene during aging was caused by a decrease in Ezh2 protein recruitment to that locus (rather than a decrease in cellular levels). Results of ChIP analysis show that in old kidneys, levels of Ezh2 protein along the locus were actually higher than those of young kidneys (Supplementary Figure S2), a difference which is not statistically significant. At Cdkn2a gene, a site with previously-described high levels of Ezh2 protein, which serves as a positive control [19], aging was also associated with increased Ezh2 density (not shown). To estimate enzymatic efficiency of Ezh2 along the Lamc1-Lamc2 locus, we divided H3K27m3 density by Ezh2 level. The H3K27m3/Ezh2 ratio was lower in old animals along the length of the entire locus (Supplementary Figure S3).

### 5mC changes at Lamc2 in aging kidneys

**Figure 4:**
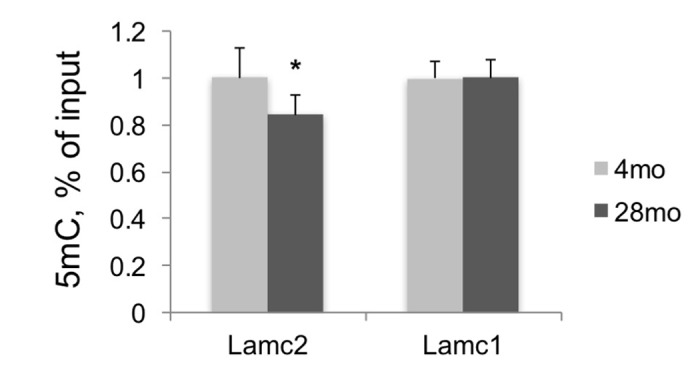
**DNA methylation analysis of Lamc2 and Lamc1 promoters**. MeDIP was done in DNA samples purified from F344 young and old kidneys. Shown normalized (to 4mo) mean values +SD, n=6 per age group, *p<0.05, **p<0.005.

5mC changes during aging were described in different organisms [21–23], and 5mC levels measured at several CpG loci predict human age with very high precision [24,25]. Therefore, along with histone modification changes in aging kidneys, we also estimated 5mC levels at Lamc2 and control genes in DNA purified from the same tissues by using MeDIP assay. This analysis revealed significant losses of 5mC at the 5’ end of Lamc2 gene but no changes at the 5’ end of Lamc1 gene, in old compared to young kidneys (Figure 4). Next, we examined the functional significance of H3K27m3 and 5mC losses for Lamc2 expression.

### The effect of Ezh2 and Dnmt inhibitors on Lamc2 expression in kidneys cells in vitro

The inverse correlation between Lamc2 mRNA levels and H3K27m3/5mC density at the gene (Figure 2D and Figure 3) suggested that these modifications mediate the silenced state of this gene, and that the increase in Lamc2 expression during aging is caused, at least in part, by the loss of H3K27 methylation and/or 5mC density at the gene. To test this suggestion, we first used an unspecific Ezh2 inhibitor, 3-deazaneplanocin (DZNep) [26]. This compound is a potent inhibitor of S-adenosylhomocysteine (AdoHcy) hydrolase [27,28]. Accumulated AdoHcy, in turn, is a competitive inhibitor of methyl-transferases, including those specific to histones and most likely DNA. It has been shown that DZNep in micromolar concentrations specifically inhibits methylation of H3K27 and activates PRC2-silenced genes [26,29], whereas its effect on DNA methylation has not been reported. Therefore, we used this inhibitor to examine its effect on H3K27m3 levels and Lamc2 expression *in vitro*.

**Figure 5:**
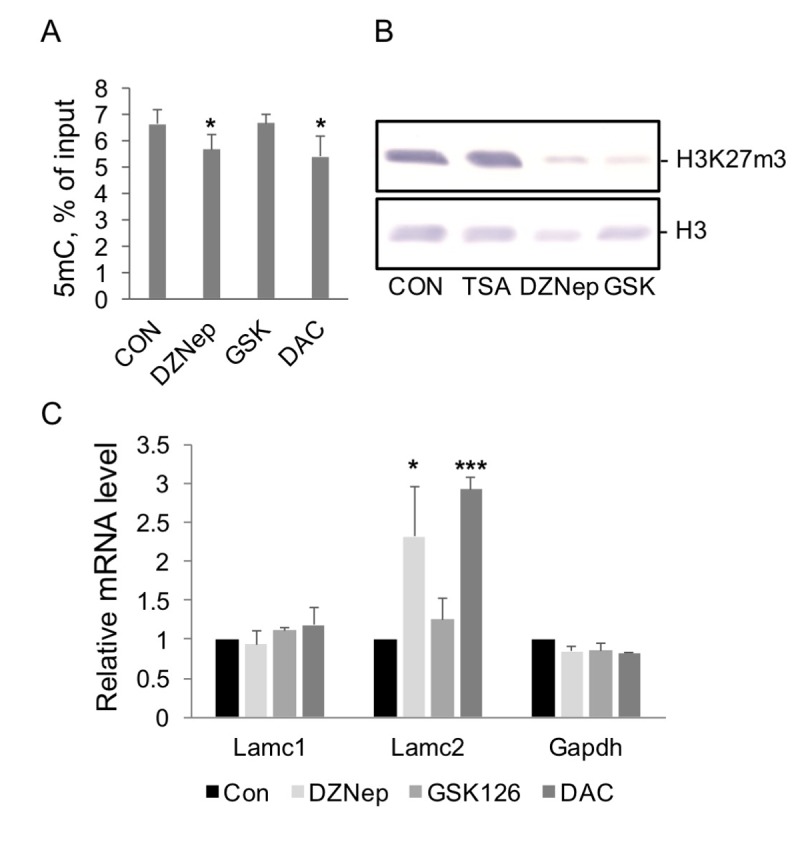
**The effect of histone modification and DNA methylation enzyme inhibitors on levels of corresponding modifications and gene transcription in HEK293 cells.** (**A**) MeDIP analysis of 5mC levels at Lamc2 gene promoter in DNA purified from cells treated with DZNep (5µM), GSK126 (2.5 µM), or DAC (5µM) for 48 hrs. Mean +SD, n=2 independent experiments, *p<0.05 compared to control. (**B**) Western blot analysis. Cells were treated with corresponding inhibitors for 48 hrs, and histones were extracted from nuclear pellets. Results of representative Western blot analysis with antibodies to H3K27m3, upper panel, and to histone H3, lower panel, are shown. (**C**) RT PCR analysis of Lamc2 and control mRNA levels in control cells (CON) and cells treated with DZNep (5µM), GSK126 (2.5 µM), or DAC (5µM) for 48 hrs. Mean +SD, n=4 independent experiments, *p<0.05, ***p<0.0005 compared to control.

In DZNep experiments we used human kidney cells (HEK293) where Lamc2 gene is not expressed. Treatment with 5µM DZNep for 48 hours slowed down the cell division rate but had little effect on cell survival (not shown). Western blot analysis revealed a decrease in global H3K27m3 levels in cells treated with DZNep compared to the controls (Figure 5). In agreement with previous observations [26,29], little or no changes were found in H3K9m3 levels at Lamc2 gene in DZNep-treated cells, indicating a selective effect on H3K27m3 histone modification (Supplementary Figure S4). RT qPCR analysis revealed substantial increases in Lamc2 transcript level after DZNep treatment, whereas no changes were observed in control Lamc1 and Gapdh transcript levels (Figure 5C).

Next, we used a more specific inhibitor of Ezh2, GSK126 [30]. This compound substantially decreases cellular levels of H3K27m3 in cells (Figure 5B). However, unlike DZNep, GSK126 treatment has no effect on Lamc2 expression (Figure 5C). These results indicate that H3K27m3 plays little or no role in the maintenance of Lamc2 silencing; therefore, loss of this modification does not result in gene upregulation. We conclude that DZNep treatment increased Lamc2 expression *in vitro* through inhibition of other than Ezh2 methyl-transferase(s). DNA methylation, a likely candidate, was tested next.

5-aza-2′-deoxycytidine, DAC, is a selective inhibitor of DNA methyl transferases [31]. DAC exposure has also been shown to substantially shorten the lifespan of cells [32]. HEK293 treatment with DAC for 48 hrs decreased DNA methylation levels at Lamc2 gene (Figure 5A) and activated its transcription to the same extent as aging *in vivo* (compare Figure 5C). Consistent with our suggestion of the role of 5mC loss in the upregulation of Lamc2, DZNep treatment significantly decreased 5mC levels at Lamc2 gene, whereas GSK126 had no effect (Figure 5A).

## DISCUSSION

We found that expression of ECM laminin genes was dysregulated during aging. In F344 and FBN-F1 rat kidneys, transcription of three genes that are expressed at very low levels in young animals – Lama3, Lamb3, and Lamc2 – progressively increased with age. These genes encode subunits of laminin 5 hetero-trimer (Ln5, laminin 332, kalinin), a major component of epidermal basement membranes and a marker of the wound healing process [33–35]. Increased expression of these genes may contribute to the development of glomerulosclerosis and tubulointerstitial fibrosis in old kidneys [6,7]. We present evidence that the loss of epigenetic gene silencing is responsible, at least in part, for the upregulation of these genes in aging kidneys.

The transcription rate of a gene depends on chromatin structure (which determines promoter accessibility) and on the availability or activity of suitable transcription factors [36,37]. Chromatin modifications that regulate transitions between states accessible or closed to transcription were examined. We found that aging in rats was associated with the loss of H3K27m3 histone modification at upregulated renal genes (Figure 1 and 2). This observation is consistent with previous studies in other model systems of aging. For example, a substantial global and gene-specific loss of H3K27m3 modification was observed in fibroblasts derived from patients with Hutchinson-Gilford progeria syndrome (premature aging) compared to controls [17]. Similarly, in aging mouse pancreatic β cells *in vivo*, there was a decline in global H3K27m3 levels and de-repression of Ink4a/Arf locus [20]. Also, in cells during senescence *in vitro*, H3K27m3 levels were reduced [19]. In contrast, our data show that, despite decreases in H3K27m3 density at upregulated genes (Figure 2B), there were little or no changes (F344), or even an increase (FBN-F1), in the total cellular levels of this modification in old kidneys, as estimated by Western blot analysis (Figure 2E). This observation indicates that, along with decreases at some sites, there must be increases in H3K27m3 density at other genomic sites; that is, it is re-distribution rather than loss of this silencing modification that takes place during aging in rat kidneys.

It has been previously shown that cellular Ezh2 protein levels decrease with aging [17,19,20]. However, we found that kidney levels of Ezh2 were instead increased in F344 old compared to young kidneys, and that levels were unchanged in longer-lived FBN-F1 animals (Figure 4). Higher levels of Ezh2 protein in shorter-lived F344 than in FBN-F1 animals parallels observations made in the Drosophila model system, where inverse correlation between the life span and cellular levels of E(z) protein has been reported [38]. Furthermore, there is no evidence of loss of Ezh2 protein at the loci where H3K27m3 levels were reduced in old animals (Supplementary Figure S1). It is therefore plausible that changes in H3K27m3 demethylases, Kdm6a/b, play a role in loss of aging-related genes. A role of Kdm6 in aging is supported in other model systems. For example, in *C. elegans,* decreased activity of Utx-1 gene (ortholog of the mammalian Kdm6a) increases life span [39,40]. Similarly, in mammalian cells *in vitro*, it has been shown that another H3K27 demethylase, Kdm6b, plays a role in the activation of PRC2-repressed loci during oncogene- and stress-induced senescence [41,42].

However, a link between the loss of H3K27m3 modification and Lamc2 gene upregulation was not corroborated in *in vitro* studies (Figure 5). Like in kidneys, Lamc1 was well expressed in HEK293 cells, whereas expression levels of Lamc2 and Nmnat2 were very low. While treatment of kidney cells with DZNep increased Lamc2 expression, the selective Ezh2 inhibitor had no effect. Taken together, these data do not support the notion that the H3K27m3-based system of gene silencing is directly involved in maintaining the silenced state of Lamc2 gene, and that this system fails during aging *in vivo*. The role of H3K27m3 loss in aging kidney remains to be determined.

The extent of cytosine methylation (5mC) of the promoter region is an important determinant of gene expression, with higher levels of methylation associated with transcriptional repression. Aging-associated changes in DNA methylation were described in different species [21–23], and changes in several CpG sites allow age prediction in humans with very high precision [24,25]. A causal relationship between DNA methylation and aging-induced gene dysregulation has been less explored. Our findings demonstrate that decreased 5mC levels at Lamc2 gene cause upregulation of this gene *in vitro* in a kidney cell line. These data suggest that in aging kidney *in vivo*, loss of 5mC at gene promoters induces transcription of these genes, at least in some cell types. Preventing or accelerating 5mC loss during aging *in vivo* will help to understand the functional significance of DNA methylation changes in aging kidneys.

One limitation of these studies is that data interpretation of ChIP-based chromatin analysis in a whole tissue is complicated by the multicellular nature of tissue, where the contribution of each cell type is not known. This issue is hard to tackle due to the lack of efficient approaches for quantitative chromatin analysis in single cells. Also, the HEK293 cell line used in these studies does not fully reflect renal cell states *in vivo*. Therefore, the effect of H3K27m3 and 5mC loss on Lamc2 expression need to be examined in all renal cell types *in vivo*. Nonetheless, the finding that H3K27m3 loss does not alter Lamc2 gene expression in one cell line suggests that this gene is not repressed by PRC2 in other cells.

In sum, these data show that, during kidney aging, there is a decrease in H3K27m3 and DNA methylation levels at silenced laminin genes associated with loss of gene silencing. *In vitro* experiments support the notion that aging-induced transcription of Lamc2 is caused, at least in part, by the loss of 5mC at the gene, whereas the role of H3K27m3 loss is less clear. Yet, Ezh2 has been implicated in gene dysregulation in other model systems of aging [17,19,20]. It is possible that different epigenetic factors mediate gene dysregulation in different species or different cell types. It is also possible that H3K27m3 density alterations at genes are secondary to transcription changes driven by other factors, as has been recently shown in mammalian system [43,44]. We suggest that the change in 5mC levels is the primary consequence of aging that alters gene transcription, whereas changes in H3K27m3 are secondary.

## MATERIALS AND METHODS

### Animal tissues

Kidneys from ad lib fed F344 (4, 24, and 28 months old, mo) and FBN-F1 (4 and 32 mo) males were used in these studies. Frozen tissues were received from the National Institute of Aging rodent tissue collection and stored at -80°C. Six animals in each group were examined. For further analysis, frozen kidneys were cut into radial segments (~1/8 part of the entire organ) while kept on liquid nitrogen. From each kidney, three fragments similarly representing major kidney compartments, cortex and medulla, were used in protein, RNA and ChIP analyses, respectively.

### Cell culture experiments

Human kidney cell line HEK293 was used in these studies. Cells were grown in DMEM supplemented with glutamine, penicillin, streptomycin, and 10% fetal bovine serum. Cells were treated with inhibitors as specified in figure legends. RNA purification, reverse transcription, and MeDIP assays were done as described below for kidney tissue.

### Western blot analysis

Cytoplasmic, nuclear and histone-enriched protein fractions were purified from kidney tissue fragments as described in [45]. Protein concentration was measured using the Bradford method (Pierce). Equal amounts of protein were loaded on 10% SDS gels for detection of Ezh2 protein, or 13% gels for histone modification analysis. Proteins were transferred on PVDF membrane. After blocking with TBST buffer (Tris-HCl, pH 7.5, 10mM; NaCl, 150 mM; Tween 20, 0.05%) containing 5% BSA, membranes were incubated with indicated primary antibodies in TBST/1% BSA buffer for one hour at room temperature, or overnight at 4°C. After washes, secondary antibodies conjugated to alkaline phosphatase (Bio Rad) were added for 1 hour at room temperature. Membranes were washed with TBST and developed with BCIP/NBT substrate. Antibodies to H3K27m3 were from Millipore; others were as listed below.

### Reverse transcription

For transcript level analysis, RNA purified from rat kidneys was treated with RNase-free DNase I, 1 U per 10 µg of RNA in 20 µl reactions, (Epicentre Technologies, Madison, WI) for 15 min at 37°C, deproteinized with phenol/chloroform mixture and precipitated with ethanol. One microgram of DNA-free RNA was reverse transcribed by Superscript II (100 U, Invitrogen, Gaithersburg, MD.) with random hexanucleotide primer mixture (1µM) in 10-µl final volume for 1 hr at 42°C. Reaction was stopped by mixing with 90µl TE (Tris-HCl 10mM, pH8.0, EDTA 1mM) and incubation at 95ºC for 5 min. RT mixtures were further diluted ten times with TE and analyzed by real time PCR with gene-specific sets of primers. Transcript levels were normalized to Gapdh mRNA levels.

### Chromatin immunoprecipitation, MeDIP, and real-time PCR analysis

ChIP was done in 96-well plates as previously described [15]. The following antibodies were used, to RNA-polymerase II (anti-CTD, clone 4H8, or anti-Pol II N terminus, both Santa Cruz), histone H3 (ab1791, Abcam), H3K9m3 (ab8898, Abcam), H3K27m3 (ab6002, Abcam; 07-449, Millipore), H3K9/14Ac (Diagenode), Ezh2 (39933, Active Motif), Kdm6a (ab36938, Abcam). Mock IP was done without added antibodies. Precipitated DNA was purified in 100μl final volume. Input DNA was purified from 10% of the amount of tissue extract used in IPs. ChIP assays were repeated at least three times. Chromatin analysis with Ezh2 antibodies was done using Fast ChIP protocol [46]. Histone modification and Pol II levels at DNA sites of interest were calculated as detailed in [15]. ChIP data were expressed as a percent of input DNA. Results of ChIP and gene expression analyses were evaluated statistically by using analysis of variance with age (young versus old) as experimental factor.

5mC antibodies used in MeDIP are specific to 5-methylated cytosines in a single stranded DNA; thus, before immuno-precipitation, DNA from animal tissues was melted by boiling. Specifically, DNA purified from cells/tissues was diluted to 0.5 ml with TE buffer, treated with ultrasound for 30 sec (Bronson sonifier, equipped with a microtip), precipitated with ethanol, washed once with 70% ethanol, dried, and dissolved in 10 μl of TE buffer. Before immunoprecipitation (IP), DNA samples were boiled for 5 min, and chilled on ice. 0.5 μg of DNA was used in one IP reaction. MeDIP was done in 96-well plates as described [47,48]. Monoclonal antibodies to 5mC (Mouse clone 33D3, Aviva, San Diego, CA), 0.3 μg per IP reaction, were used. Mock IP was done without added antibodies. qPCR analysis of precipitated DNA was done with gene-specific primers. 10% of the amount of input DNA used in IPs was analyzed in parallel by qPCR to estimate efficiency of IP.

PCR reaction mixture contained 2.5μl 2X SYBR Green PCR master mix (SensiMix, Quantace), 2μl DNA template and 0.2μl primers (10 μM each) in 5μl final volume in 384-Well Optical Reaction Plate (Applied Biosystems). Amplification (three steps, 40 cycles), data acquisition and analysis were carried out using the 7900HT Real Time PCR system and SDS Enterprise Database software (Applied Biosystems). PCR reactions were run in triplicate. Standard dilutions of genomic DNA (for genomic targets) or dilutions of pooled RT reactions (for cDNA targets) were included in each PCR run. Sequences of primers used in these studies are available upon request.

## Supplementary Material

Supplementary File
