# Supplementary material for: Chromatin changes trigger laminin genes dysregulation in aging kidneys
Source: Aging (Albany NY). 2018 May 29;10(5):1133–45. doi: 10.18632/aging.101453 (PMC5990391; doi:10.18632/aging.101453)
Supplement: Supplementary File [file aging-10-101453-s001.pdf]

SUPPLEMENTARY MATERIAL

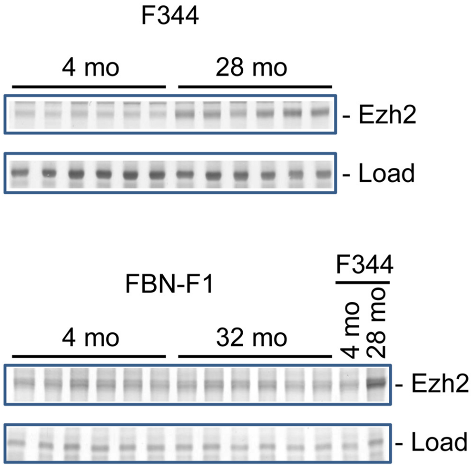

Figure S1. Western blot analysis of Ezh2 protein levels in whole cell extracts from young and old F344 and FBN-F1 kidneys. Equal amounts of extracted proteins from tissue fragments were subjected to SDS PAGE in 10% gels, transferred to PVDF membranes and stained with anti-Ezh2 antibodies. Representative parts of ponseau-stained membranes are also shown (Load).

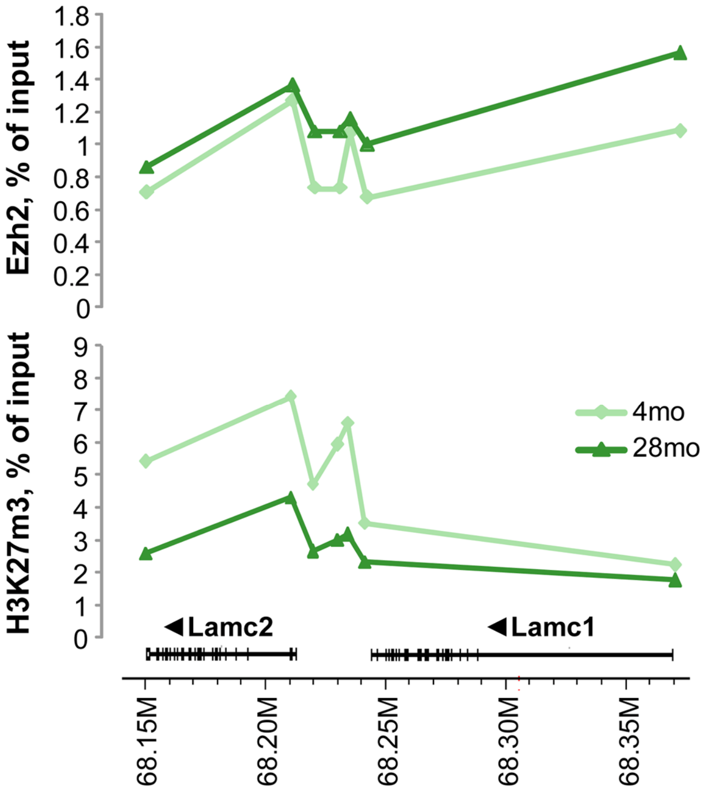

Figure S2. Ezh2 density along the Lamc2-Lamc1 locus. ChIP analysis with Ezh2 (Upper panel) and H3K27m3 (Lower panel) antibodies was done in young and old kidney extracts. Chromosome 13 coordinates (in million base pairs, M) are shown below. Ezh2 and H3K27m3 ChIP signals are shown as percent of input, n=6 per age group, mean values are presented.

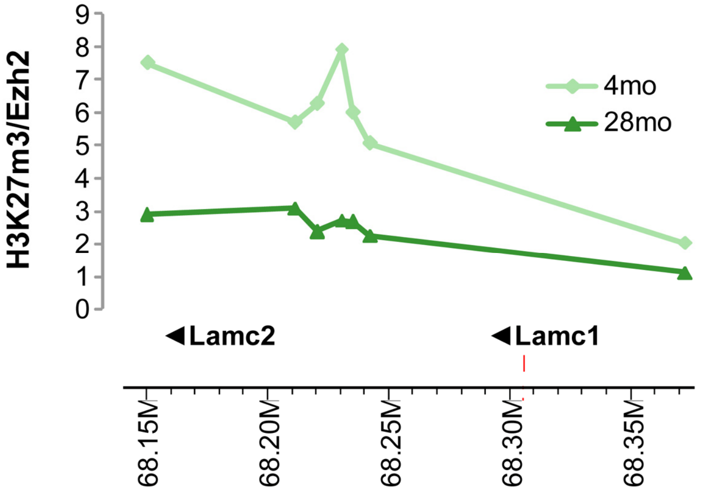

Figure S3. Changes in H3K27m3/Ezh2 ratio along the Lamc2-Lamc1 locus. Mean values shown in Supplemental Figure 2 were used to calculate the ratio.

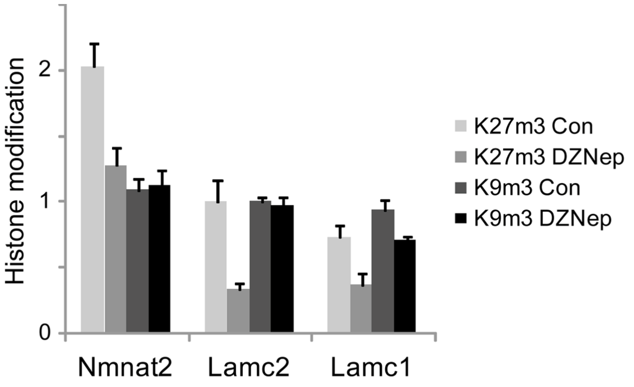

Figure S4. ChIP analysis of H3K27m3 and H3K9m3 density at the promoters of Nmnt2, Lamc2, and Lamc1 genes in untreated (Con) and DZNep treated (5 µM, 48 hrs) cells. ChIP was done in cell extractes as described in Figure 1. Data are expressed as percent of input, mean±SD values of two independent experiments are shown.
